# Supplementary material for: Aggressive dominance can decrease behavioral complexity on subordinates through synchronization of locomotor activities
Source: Commun Biol. 2019 Dec 12;2:467. doi: 10.1038/s42003-019-0710-1 (PMC6908596; doi:10.1038/s42003-019-0710-1)
Supplement: Supplementary file 1 — Supplementary Information [file 42003_2019_710_MOESM1_ESM.pdf]

Supplementary Figures

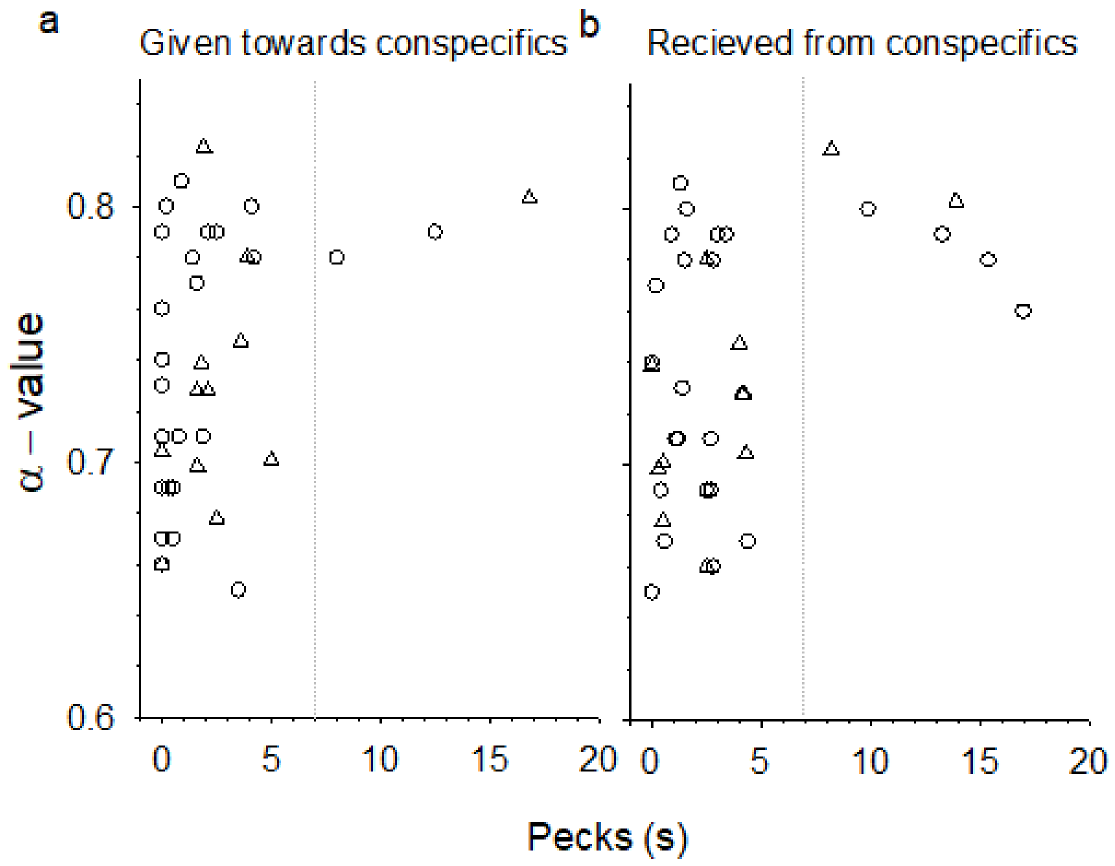

Supplementary Figure 1. The complexity of ambulatory dynamic as a function of aggressive pecking behavior during the first hour of testing in social groups (day 1).

The complexity of ambulatory dynamic as a function of a) the time spent performing aggressive pecks towards both conspecifics and b) the time receiving pecks from both conspecifics during one-hour period. Females (open circles) and males (open triangles) that performed or received either more than 6.5s of pecks dotted line) all showed α-values above 0.76. The α-values were estimated with the locomotor time series using DFA3 (see Materials and Methods

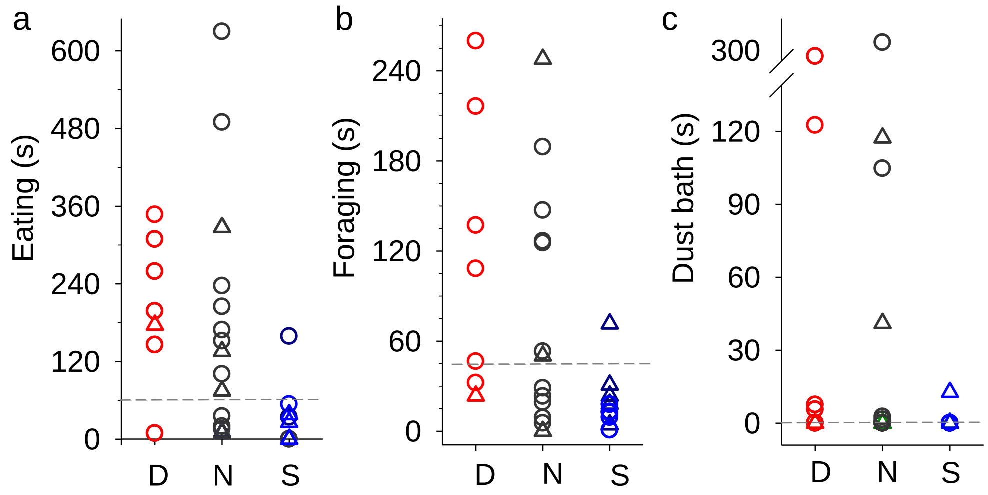

Supplementary Figure 2. A higher proportion of dominant (D) and neutral (N) birds spend larger times eating, foraging and dust bathing than the subordinate (S) counterparts after 48 hours of group-forming.

Females (open circled) and males (open triangles). According to pecking behavior, birds were classified as Dominant (D), Subordinate (S) or belonging to a neutral (N) group. In order to better evidence differences between groups, an arbitrary threshold of 60, 45 and 0 s performing respectively eating ("a"), foraging ("b") and dust bathing ("c") were drawn (grey dashed lines).

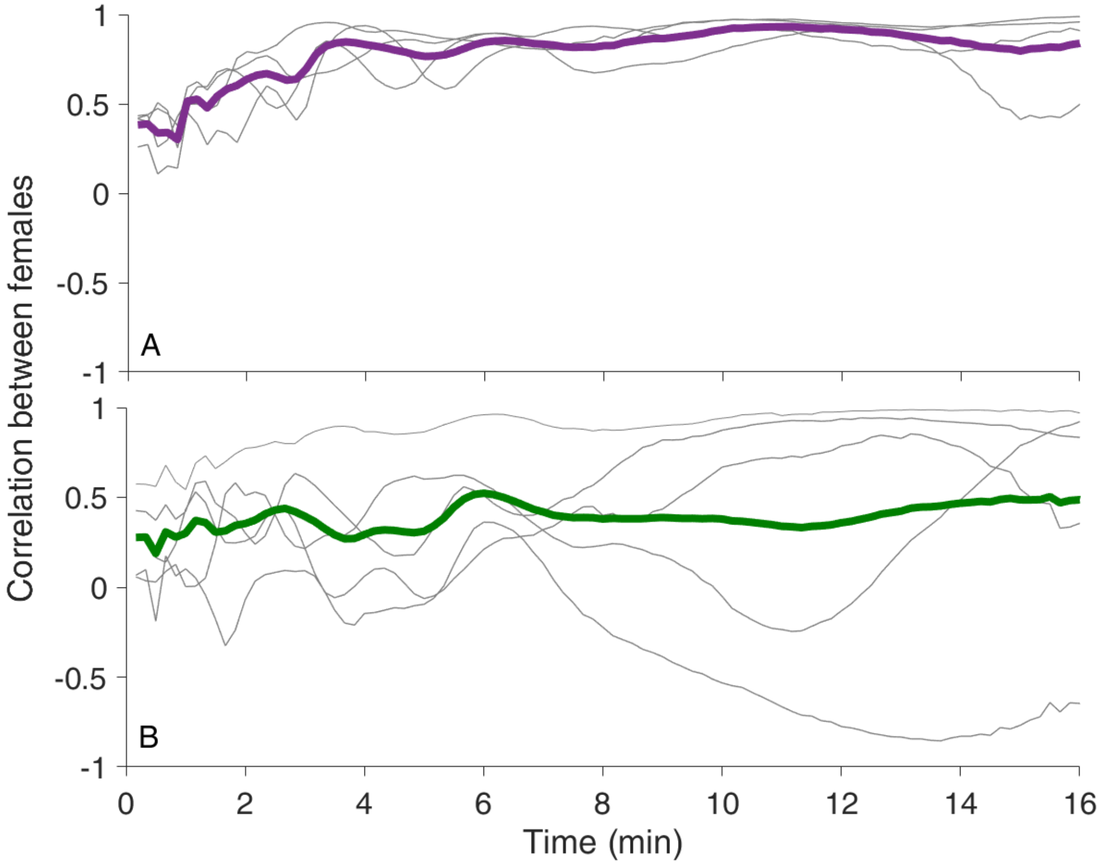

Supplementary Figure 3. Pairwise comparison of the real part of wavelet coefficients (grey lines) between A) the subordinate and the dominate female and B) the two neutral females within the social group at each time scale was performed using the Spearman correlation coefficient. Mean values are shown in thick line within each panel, in violet and dark green respectively. Wavelet analysis was performed on actograms with 15 s bins in order to reduce noise.

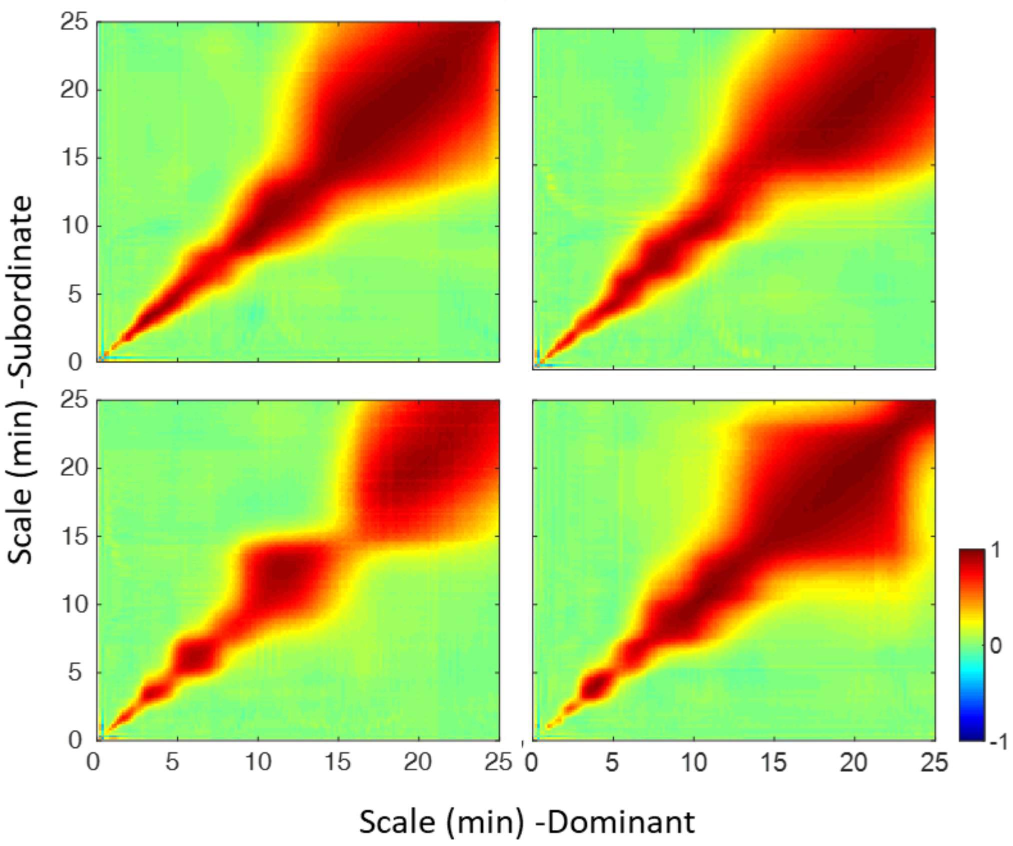

Supplementary Figure 4. The four panels represent each of the four social groups where clear dominate-subordinate relationships are observed. Within each panel, the pairwise comparison of the real part of wavelet coefficients between the subordinate and the dominant female within each social group for the full range of time scale is shown. Comparison was performed using the Spearman correlation coefficient. Grey lines in Supplementary Figure 3A are the corresponding diagonal values of each panel. Wavelet analysis was performed on actograms with 15 s bins in order to reduce noise.

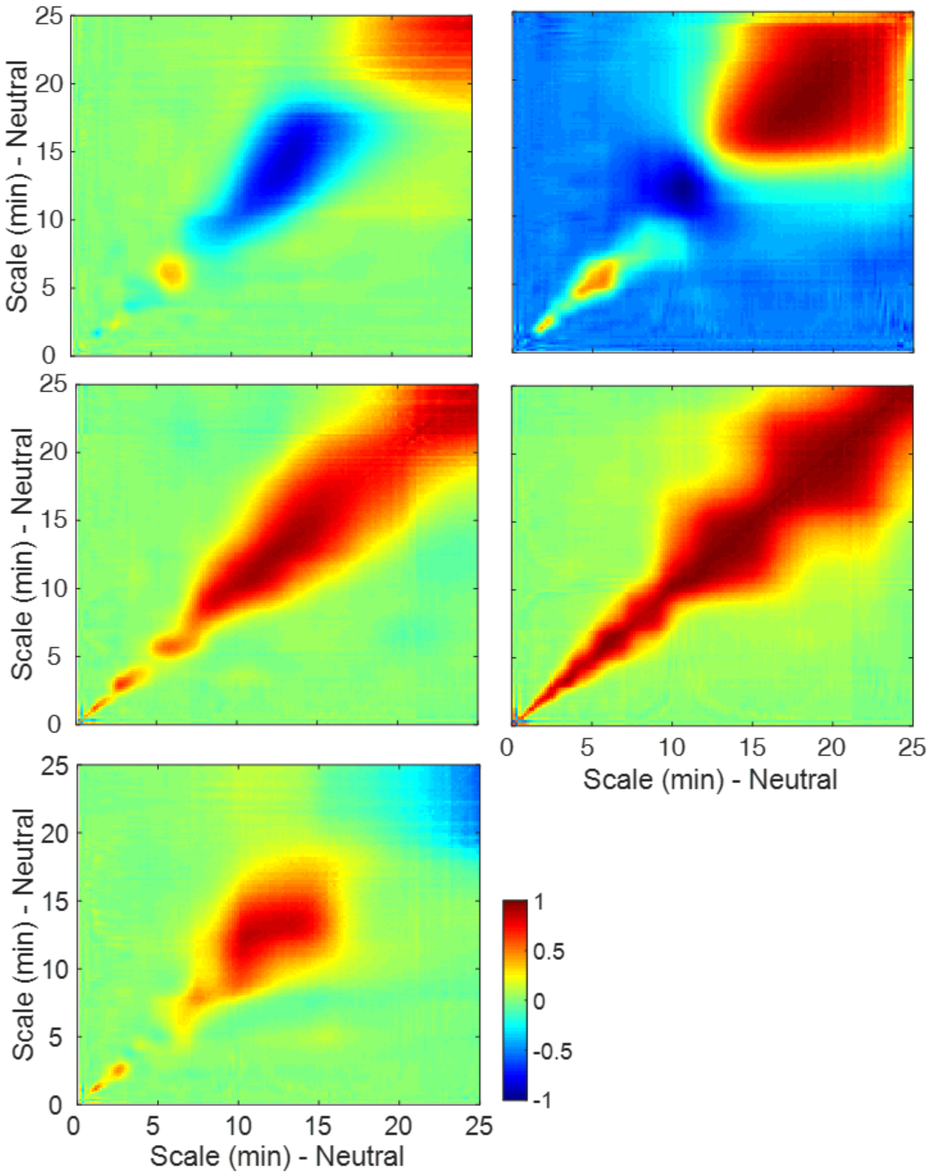

Supplementary Figure 5. The five panels represent each of the five social group with all neutral members. Within each panel, the pairwise comparison of the real part of wavelet coefficients between the female within neutral social group for the full range of time scale is shown. Comparison was performed using the Spearman correlation coefficient. Grey lines in in Supplementary Figure 3B are the corresponding diagonal values of each panel. Wavelet analysis was performed on actograms with 15 s bins in order to reduce noise.

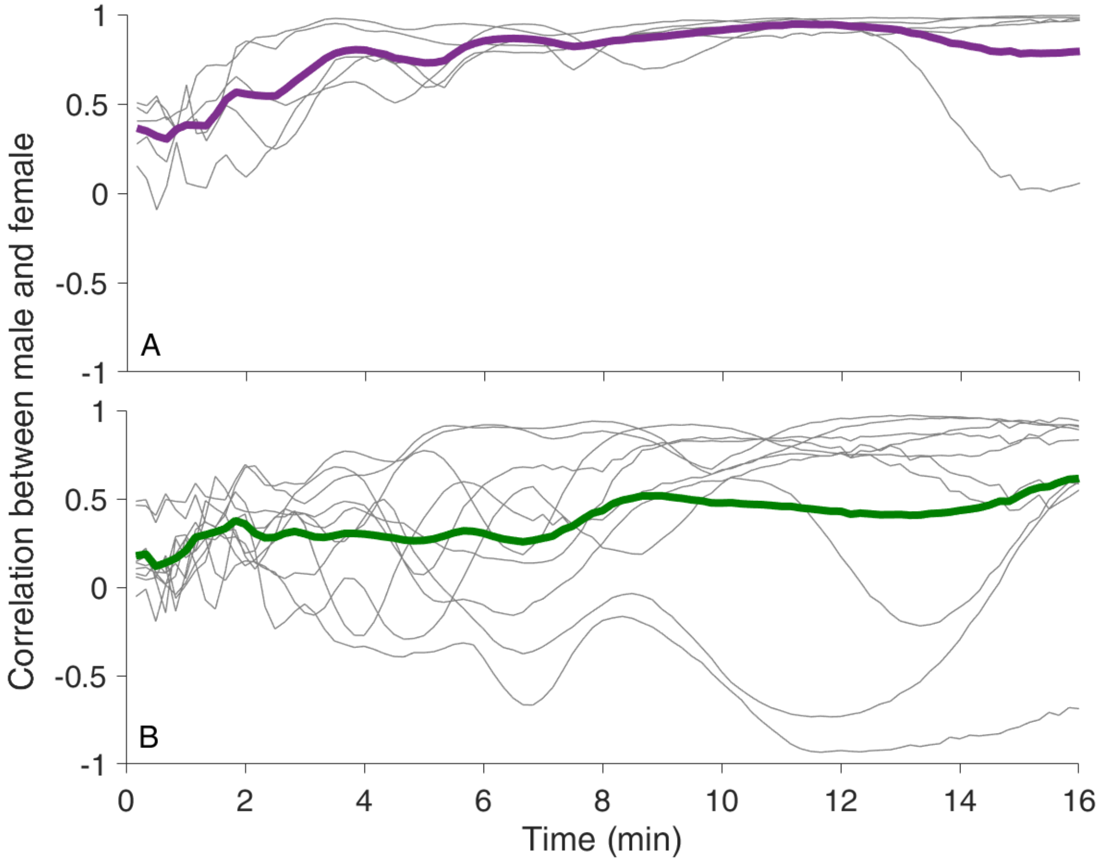

Supplementary Figure 6. Pairwise comparison of the real part of wavelet coefficients (grey lines) between A) the subordinate male and the dominate female and B) the neutral male and each of the two neutral females within the social group at each time scale was performed using the Spearman correlation coefficient. Mean values are shown in thick line within each panel, in violet and dark green respectively. Wavelet analysis was performed on actograms with 15 s bins in order to reduce noise.

Supplementary Tables

Supplementary Table 1. Individual female dyadic pecking interactions with both male and female conspecifics in the social group.

Social group	Female ID	Duration (s) of interaction with male			Duration (s) of interaction with female			Clasification	α-value
		Performed towards	Recieved from	Aggressive Score*	Performed towards	Recieved from	Aggressive Score*		
9	1643	2.4	0	2.4	18.2	0	**18.2**	Dominate	0.86
10	1195	17.3	0	**17.3**	1.1	0	1.1	Dominate	0.85
2	6258	16.3	0	**16.3**	14.5	0	**14.5**	Dominate	0.87
5	1178	9.5	0	**9.5**	10	0	**10**	Dominate	0.83
11	4500	6.6	0	**6.6**	6.5	0	**6.5**	Dominate	0.77
6	4232	5.1	0.2	**4.9**	1.5	0	1.5	Dominate	0.79
7	4237	3.6	0.2	3.4	2.6	0	2.6	Neutral	0.86
3	4290	2	0	2	2.2	0	2.2	n/d	0.88
1	1642	0.5	0	0.5	4.8	0	4.8	Neutral	0.79
8	4271	0.6	0	0.6	0.5	0	0.5	Neutral	0.74
4	4238	0.5	0.3	0.2	0	0	0	Neutral	0.78
4	4295	0	0	0	0	0	0	Neutral	0.79
12	4280	0	0	0	0	0.2	-0.2	Neutral	0.76
12	4278	0	0.5	-0.5	0.2	0	0.2	Neutral	0.82
8	4230	0	0	0	0	0.5	-0.5	Neutral	0.70
10	1181	0	0	0	0	1.1	-1.1	n/d	0.76
6	6268	0	0	0	0	1.5	-1.5	n/d	0.71
7	4300	0	0.3	-0.3	0	2.6	-2.6	Neutral	0.87
1	6289	2.3	0.3	2	0	4.8	-4.8	Neutral	0.85
11	4264	0	0.6	-0.6	0	6.5	**-6.5**	Subordinate	0.93
5	1196	0.3	0.2	0.1	0	10	**-10**	Subordinate	1.03
3	6280	0	11.3	**-11.3**	0	2.2	-2.2	Subordinate	0.97
2	1677	0	0	0	0	14.5	**-14.5**	Subordinate	0.94
9	6283	0	0	0	0	18.2	**-18.2**	Subordinate	0.93

Aggressive score is the difference between the time spent performing pecking towards and time receiving pecks from conspecific during dyadic interacting within a social group (1male and 2 females)
Red values indicate the top 20% of individuals (dominate) with the highest agonistic score which corresponded to scores ≥ 4.9s, and in blue the respective subordinate birds with aggressive scores ≤-4.9. Neutral represents birds where dominate birds were not detected in their social group. When none of the above criteria were observed animals were classified as non-determined (n/d)

Supplementary Table 2. Male dyadic pecking interactions with both female conspecifics in the social group.

Social group	Male ID	Duration (s) of interaction with female			Duration (s) of interaction with female			Clasification	α-value
		Performed towards	Recieved from	Aggressive Score*	Performed towards	Recieved from	Aggressive Score*		
3	4254	0	2	-2	11.3	0	**11.3**	Dominate	0.81
12	4499	0	0	0	0.5	0	0.5	Neutral	0.71
4	4299	0	0	0	0.3	0.5	-0.2	Neutral	0.72
8	4249	0	0.6	-0.6	0	0	0	Neutral	0.78
1	6287	0.3	2.3	-2	0	0.5	-0.5	Neutral	0.75
9	6252	0	0	0	0	2.4	-2.4	n/d	0.72
7	4281	0.3	0	0.3	0.2	3.6	-3.4	n/d	0.77
6	6278	0.2	5.1	**-4.9**	0	0	0	Subordinate	0.93
11	4273	0	6.6	**-6.6**	0.6	0	0.6	Subordinate	0.96
5	1684	0	9.5	**-9.5**	0.2	0.3	-0.1	Subordinate	0.97
2	1172	0	16.3	**-16.3**	0	0	0	Subordinate	0.96
10	6288	0	17.3	**-17.3**	0	0	0	Subordinate	0.93

*Aggressive score is the difference between the time spent performing pecking towards and time receiving pecks from conspecific during dyadic interacting within a social group (1male and 2 females)
Red values indicate the top 20% of individuals (dominate) with the highest aggressive score which corresponded to scores ≥ 4.9s, and in blue the respective subordinate birds with agonistic scores ≤-4.9. Neutral represents birds where dominate birds were not detected in their social group. When non of the above criteria were observed animals were clasified as non-determined (n

Supplementary Table 3. Correlations between behavioral variables analyzed in female quails within small social groups.

	Pecks towards female	Chase towards female	Pecks towards male	Chase towards male	Pecks recieved from female	Chase recieved from female	Pecks recieved from male	Chase recieved from male	Grabs	Mounts	Cloacal contacts	Eating	Drinking	Foraging	Dust bath	Ambulation	α-value
Pecks towards female	1																
Chase towards female	0.92	1															
Pecks towards male	0.8	0.83	1														
Chase towards male	0.9	0.96	0.89	1													
Pecks recieved from fem.	-0.75	-0.75	-0.62	-0.75	1												
Chase recieved from fem.	-0.75	-0.75	-0.61	-0.75	0.92	1											
Pecks recieved from male	-0.33	-0.29	-0.19	-0.3	0.25	0.26	1										
Chase recieved from male	0.01	0.03	0.08	0.05	-0.14	-0.29	0.3	1									
Grabs	-0.39	-0.4	-0.33	-0.35	0.23	0.33	0.26	0.11	1								
Mounts	-0.57	-0.55	-0.56	-0.55	0.26	0.42	0.28	-0.08	0.86	1							
Cloacal contacts	-0.49	-0.38	-0.46	-0.38	0.31	0.43	0.27	-0.1	0.63	0.78	1						
Eating	0.31	0.44	0.37	0.49	-0.41	-0.44	-0.12	0.1	-0.15	-0.35	-0.42	1					
Drinking	0.43	0.42	0.22	0.43	-0.48	-0.5	-0.26	-0	0.05	-0.1	-0.26	0.71	1				
Foraging	0.43	0.5	0.58	0.5	-0.46	-0.44	-0.37	-0.15	-0.44	-0.46	-0.48	0.28	0.17	1			
Dust bath	0.35	0.37	0.58	0.48	-0.37	-0.44	-0.15	0.1	-0.25	-0.43	-0.39	0.3	0.11	0.57	1		
Ambulation	0.23	0.13	0.23	0.09	0.12	0.19	0.12	0.04	-0.31	-0.4	-0.28	-0.05	-0.2	0.01	-0.07	1	
α-value	-0.1	-0.14	-0.02	-0.11	0.46	0.41	0.46	0.08	-0.03	-0.03	0.18	-0.28	-0.3	-0.39	-0.28	0.31	1

Supplementary Table 4. Eigenvalues of principal component analysis

	Females Eigenvalues		Males Eigenvalues	
	e1	e2	e1	e2
**Foraging (s)**	0.59	0.16	0.48	-0.46
**α-value**	-0.52	0.37	0.42	0.58
**Eating (s)**	0.49	0.06	-0.48	-0.08
**Pecks performed (s)**	0.32	0.63	-0.16	0.17
**Ambulation (%)**	-0.20	0.66	0.52	-0.33
**Grabs (s)**	-	-	-0.25	-0.56

Supplementary Methods

Behavioral tests

Partial mechanical restraint test was performed at 40 days of age. This test consists in restricting the movement of the animal between two walls of a melamine box of 20 x 10 cm (height per width, respectively) with the characteristic that the front wall was made of glass (it allows the visualization of the animal and video recording of its behavior), and the back wall was adjustable to induce immobility in such a way that the animal cannot open the wings, but can move the head and legs (Jones et al 2000). The experimenters retreated out of the birds' sight, and the test was during 5 minutes recorded with a video camera place in front of the box. All the birds were tested in 31 batches of 4 animals each, where the birds had no visual or physical contact between each other. The video was analyzed manually, and the following variables were recorded: the latency to struggle: time between the initiation of restraint until the first struggling episode (defined as the movement of the legs of the animal trying to escape) and the number of struggles during the observation period. The struggle episodes were considered different if they were separated by 5 s or more. The immobility of the animal during the test has been widely considered in the literature as an indicator of intense fear (*1-3*). Those whose latency of struggle was> 100s were considered as fearful (*4*).

Tonic immobility (IT) test was performed at 100 days of age. According to (*5*) this test induces an unlearned antipredator response that is triggered by a brief period of physical restraint. In the test the individual was place in the left lateral *decubitus* and hold for 15 seconds (the necessary time required to unleash the muscular immobilized tonic behavior), holding him with both hands against a support base (one hand on the head and another in the body). It was recorded the number of inductions to achieve an immobility of at least 10 seconds and the duration of the immobility (IT). A long duration of IT and a smaller number of necessary inductions is indicative of a high level of fear as opposed to a short response (*5*). If IT was not attained after 5 successive attempts, the bird was considered not to be susceptible and

scores of 0 were given for TI duration. The birds considered "fearful" required a single induction in the test, while "non-fearful" birds were selected based on those that needed more than 4 inductions.

Social interaction (SI) test was performed between 70 days of age. This test is described in detail in Caliva et al (2017) including a figure with the schematic representation of the test procedure. Briefly, the SI test consists in a 5 min encounter between an unfamiliar test adult bird and a photocastrated stimulus adult male, in the presence of the test bird's cagemate (audience). A video-camera was positioned 1 m above the apparatus and connected to a computer that allowed constant monitoring and recording during the test while out of the sight of the birds. Using behavioral tracking software (Any-maze, 2015) the following aggressive behaviors were recorded: pecks, grabs, mounts, cloacal contacts, threats, chase, and attack with claws. Herein, when grabs, mounts or cloacal contacts were performed by one male towards another male, they were considered as aggressive behaviors (*6*). Birds that performed more than 5 aggressiveness behaviors were considered aggressive, and birds that did not perform any aggressive behavior towards the photocastrated opponent were considered non-aggressive (*7*). If during the interaction a quail received more than 5 consecutive aggressive pecks, showed a clear and continued escaping (retrieval) behavior, and/or showed any sign of physical injuries testing was finalized immediately.

Welfare assessment. At 96 and 108 days of age female skin lesions and plumage status were evaluated following a procedure proposed by Pellegrini et al. (*8*) that is an adapted version of the protocol proposed by Welfare Quality ® consortium (*9*). Head, neck and back skin lesions were determined using a score scale from 0 to 2 where "0" represents no lesions (punctiform damage <0.25 cm diameter) or scratches, "1" represents less than 3 lesion or scratches, and "2" reflects 3 or more lesion or scratches. *Head, neck and back plumage damage* was also determined using a score scale from 0 to 2 as follows: "0" represents individuals with no plumage damage or slight wear (only single feathers lacking), "1" represent individuals with one or more body parts that have moderate wear (i.e. damaged feathers worn or deformed) or one or more featherless areas < 1.5 cm in diameter at the larger extent and "2"

corresponded to individuals that have at least one featherless area > 1.5 cm in diameter at the largest extent. Males induced plumage damage (score >0) to female cagemates were considered aggressive(*8*), while non-aggressive males were those in which no plumage damage was seen in female cagemates (*8*).

References

1. A. G. Moriarty, Anxiogenic effects of a β-Carboline on a tonic immobility and open field behaviour in chickens (Gallus gallus). *Pharm Bioch Behav* **51**, 795-798 (1995).
2. R. B. Jones, D. G. Satterlee, Threat-induced behavioural inhibition in Japanese quail genetically selected for contrasting adrenocortical response to mechanical restraint. *Brit Poultry Sci* **37**, 465-470 (1996).
3. J. M. Faure, R. B. Jones, W. Bessei, Fear and social motivation in open-field behaviour of the domestic chick. A theoretical consideration. *Biol Behav* **8**, 103-116 (1983).
4. J. M. Kembro, Universidad Nacional de Cordoba, (2010).
5. R. B. Jones, The tonic immobility reaction of the domestic fowl: a review. *World's Poultry Sc J* **42**, 82-96 (1986).
6. E. Adkins-Regan, Male-male sexual behavior in Japanese quail: Being "on top" reduces mating and fertilization with females. *Beh Proc* **108**, 71–79 (2015).
7. J. M. Caliva, J. M. Kembro, S. Pellegrini, D. A. Guzman, R. H. Marin, Unexpected results when assessing underlying aggressiveness in Japanese quail using photocastrated stimulus birds. *Poult Sci* **96**, 4140-4150 (2017).
8. S. Pellegrini, L. Condat, R. H. Marin, D. A. Guzman, Can Japanese quail male aggressions toward a female cagemate predict aggressiveness toward unknown conspecifics? *Livest Sci* **22**, 65-70 (2019).
9. WelfareQuality®, *Welfare Quality® Assessment protocol for poultry (broilers, laying hens)*. (Welfare Quality® Consortium, Lelystad, Netherlands, 2009).
