# Supplementary material for: Aggressive dominance can decrease behavioral complexity on subordinates through synchronization of locomotor activities
Source: Commun Biol. 2019 Dec 12;2:467. doi: 10.1038/s42003-019-0710-1 (PMC6908596; doi:10.1038/s42003-019-0710-1)
Supplement: Supplementary file 5 — Description of Additional Supplementary Files [file 42003_2019_710_MOESM5_ESM.pdf]

Description of Additional Supplementary Files

File Name: **Supplementary Movie 1**

Description: **Short movie of a social environment with a dominant individual**. All individuals are identified with a color coded number as visualized by the IdTracker software. Specifically, the dominant individual is identified with the yellow number 3, and can clearly be seen chasing the other members of the social group. Noteworthy, the movie is three-times faster than the original recording

File Name: **Supplementary Movie 2**

Description: **Short movie of a social environment without a dominant individual (Neutral group).** All individuals are identified with a color coded number as visualized by the IdTracker software. Although group members can interact they are apparently performing independent behavior, with no observable no agonistic behavior between them. Noteworthy, the movie is three-times faster than the original recording.
