# Supplementary material for: Aggressive dominance can decrease behavioral complexity on subordinates through synchronization of locomotor activities
Source: Commun Biol. 2019 Dec 12;2:467. doi: 10.1038/s42003-019-0710-1 (PMC6908596; doi:10.1038/s42003-019-0710-1)
Supplement: Supplementary file 6 — Reporting Summary [file 42003_2019_710_MOESM6_ESM.pdf]

nature research

Corresponding author(s): Jackelyn Kembro

Last updated by author(s): Apr 16, 2019

Reporting Summary

Nature Research wishes to improve the reproducibility of the work that we publish. This form provides structure for consistency and transparency in reporting. For further information on Nature Research policies, see Authors & Referees and the Editorial Policy Checklist.

Statistics

For all statistical analyses, confirm that the following items are present in the figure legend, table legend, main text, or Methods section.

n/a	Confirmed	
☐	☒	The exact sample size (*n*) for each experimental group/condition, given as a discrete number and unit of measurement
☐	☒	A statement on whether measurements were taken from distinct samples or whether the same sample was measured repeatedly
☐	☒	The statistical test(s) used AND whether they are one- or two-sided<br>*Only common tests should be described solely by name; describe more complex techniques in the Methods section.*
☒	☐	A description of all covariates tested
☐	☒	A description of any assumptions or corrections, such as tests of normality and adjustment for multiple comparisons
☐	☒	A full description of the statistical parameters including central tendency (e.g. means) or other basic estimates (e.g. regression coefficient) AND variation (e.g. standard deviation) or associated estimates of uncertainty (e.g. confidence intervals)
☐	☒	For null hypothesis testing, the test statistic (e.g. *F*, *t*, *r*) with confidence intervals, effect sizes, degrees of freedom and *P* value noted<br>*Give P values as exact values whenever suitable.*
☒	☐	For Bayesian analysis, information on the choice of priors and Markov chain Monte Carlo settings
☒	☐	For hierarchical and complex designs, identification of the appropriate level for tests and full reporting of outcomes
☒	☐	Estimates of effect sizes (e.g. Cohen's *d*, Pearson's *r*), indicating how they were calculated

Our web collection on statistics for biologists contains articles on many of the points above.

Software and code

Policy information about availability of computer code

Data collection	IdTracker is a videotracking software that keeps the correct identity of each individual during video behavioral analysis and is publicly available at http://www.idtracker.es/.<br>ANY-MAZE@ is a licensed video tracking program, that can be downloaded from http://www.anymaze.co.uk/.
Data analysis	The customized Matlab code customized code Locomotion.m in publicly available on Figshare. Kembro, J. M. Source code for: Locomotor time series from x,y IdTracker coordinates in Matlab. Figshare https://doi.org/ 10.6084/m9.figshare.7716284 (2019).

For manuscripts utilizing custom algorithms or software that are central to the research but not yet described in published literature, software must be made available to editors/reviewers. We strongly encourage code deposition in a community repository (e.g. GitHub). See the Nature Research guidelines for submitting code & software for further information.

Data

Policy information about availability of data

All manuscripts must include a data availability statement. This statement should provide the following information, where applicable:

- Accession codes, unique identifiers, or web links for publicly available datasets
- A list of figures that have associated raw data
- A description of any restrictions on data availability

All raw data and video records are publicly available and are reported in detail in our data descriptor manuscript in Sci Data (SDATA-18-00584A) recently accepted for publication

1. Caliva, J. M., Alcala, R., Guzman, D. A., Marin, R. & Kembro, J. M. Four behavioral tests associated with fear and aggressiveness in Japanese quail. figshare https://doi.org/10.6084/m9.figshare.7122926.v1 (2019).
2. Alcala, R., Caliva, J. M., Marin, R. H. & Kembro, J. M. High-resolution, 1-hour, locomotor time series of Japanese quail in diverse social environments. figshare https://doi.org/10.6084/m9.figshare.7117631.v1 (2019).
3. Alcala, R., Caliva, J. M., Marin, R. H. & Kembro, J. M. One-hour social and reproductive behavioral time series of Japanese quail in diverse social environments. figshare https://doi.org/10.6084/m9.figshare.7117679.v1 (2019).

4. Kembro, J. M., Guzmán, D., Caliva, J. M., Alcala, R. & Marin, R. H. High-resolution behavioral time series of Japanese quail within their social environment. figshare https://doi.org/10.6084/m9.figshare.c.4424327 (2019).

Field-specific reporting

Please select the one below that is the best fit for your research. If you are not sure, read the appropriate sections before making your selection.

☒ Life sciences ☐ Behavioural & social sciences ☐ Ecological, evolutionary & environmental sciences

For a reference copy of the document with all sections, see nature.com/documents/nr-reporting-summary-flat.pdf

Life sciences study design

All studies must disclose on these points even when the disclosure is negative.

Sample size	In this study, animals were preselected based on a combination of 4 behavioral tests, taking into consideration that quail that are more fearful also tend to be more aggressive. Thus half of the 12 social groups (2 females: 1 male) evaluated had birds of type A that were expected to be more fearful and aggressive, while the other half of the groups had birds of type B that were expected to be less fearful and non-aggressive. Since expectations do not necessarily align with actual results we estimated that a minimum of 4 groups of each type (with and without aggressive dominate individuals) was necessary in order to obtain a potency for analysis of > 80% and confidence level of 95%.This minimum sample size was calculated based on our recent paper Caliva et al 2017 (http://dx.doi.org/10.3382/ps/pex258) were a variance of 3.3 was estimated in expression of aggressive pecks, and a minimum difference between means of 4.5 seconds of aggressive pecks.
Data exclusions	All the data of animals tested in social groups were analized (no animals were excluded).
Replication	Since only 4 social groups (2 with type A birds and 2 with type B birds) could be tested simultaneously, the setup was repeated 3 consecutive times.
Randomization	One-hundred and six quail were randomly housed in cages. The order in which they were evaluated in each of the preselection testing was also randomized. These tests were used as a preselection criterion for social group testing, and birds were assigned to a given group based on thier preformance in preselection tests. The order of testing of each social group was randomized.
Blinding	All data analysis and technical validation was performed by one observer both in Any-Maze as well as in IdTracker. In both cases the observer was blinded regarding the prior history of the animals allocated in each group.

Reporting for specific materials, systems and methods

We require information from authors about some types of materials, experimental systems and methods used in many studies. Here, indicate whether each material, system or method listed is relevant to your study. If you are not sure if a list item applies to your research, read the appropriate section before selecting a response.

Materials & experimental systems

n/a	Involved in the study	
☒	☐	Antibodies
☒	☐	Eukaryotic cell lines
☒	☐	Palaeontology
☐	☒	Animals and other organisms
☒	☐	Human research participants
☒	☐	Clinical data

Methods

n/a	Involved in the study	
☒	☐	ChIP-seq
☒	☐	Flow cytometry
☒	☐	MRI-based neuroimaging

Animals and other organisms

Policy information about studies involving animals; ARRIVE guidelines recommended for reporting animal research

Laboratory animals	Japanese quail (Coturnix japonica)
Wild animals	Study did not involve wild animals
Field-collected samples	Study did not involve samples collected from the field
Ethics oversight	All the procedures were in compliance with the Guide for the Care and Use of Laboratory Animals issued by the National Institute of Health (NIH Publications, Eighth Edition). Experimental protocol was approved by the Institutional Council for the Care of Laboratory Animals (CICUAL, Comité Institutional de Cuidado de Animales de Laboratorio) of the Instituto de Investigaciones Biologicas y Technologicas (IIByT, CONICET - Universidad Nacional de Córdoba).

Note that full information on the approval of the study protocol must also be provided in the manuscript.
